# Evaluation of Pallet Covers Performance for Produce Protection in Cold Chain Logistics for Chard, Cucumbers and Carrots

**DOI:** 10.3390/foods12152961

**Published:** 2023-08-04

**Authors:** Ricardo Badia-Melis, Luis Ruiz-Garcia, Jose Ignacio Robla-Villalba, Pedro Hoyos-Echevarria

**Affiliations:** 1 Departamento de Ingeniería Agroforestal, ETSIAAB, Universidad Politécnica de Madrid (UPM), Edificio Motores, Avda. Puerta de Hierro 2, 28040 Madrid, Spain; 2 Sensors Technology Laboratory, Centro Nacional de Investigaciones Metalúrgicas, Consejo Superior de Investigaciones Científicas (CENIM-CSIC), Avenida Gregorio del Amo 8, 28040 Madrid, Spain; 3 Departamento de Producción Agraria, ETSIAAB, Universidad Politécnica de Madrid (UPM), Edificio Agrícolas, Avda. Puerta de Hierro 2, 28040 Madrid, Spain

**Keywords:** cold chain, pallet cover, thermal image, food control, food safety

## Abstract

Cold chain disruption and refrigeration failures are common issues in the logistics of perishable food products. In these cases, the use of pallet covers should be very useful, delaying the increase of product temperatures inside the pallets until cooling conditions are restored. However, there are no studies about the performance of pallet covers in these types of situations, which could persist for hours. This paper evaluates the performance of three different types of cold chain covers versus having no cover for three different types of vegetables (chard, cucumbers, and carrots). A refrigeration failure during the cold chain was simulated. The three covers presented an improvement in temperature loss compared to the no-cover situation, with the average time for the temperature to increase from 4 to 10 °C with a cover being a range of 214 to 506 min, while for no cover, from 162 to 211 min. Relative humidity (RH) always presented improved preservation with a cover than with no cover, except for one case. The correlation between the thermal images and sensor temperatures was also studied.

## 1. Introduction

The production, storage, distribution and transport of cold-sensitive products occur daily around the world. Thus, temperature control is essential for these products. The biggest challenge is to ensure a continuous cold chain from the producer to the consumer, and to do so, it is necessary to count on adequate monitoring and control systems [1]. Inadequate management of this supply chain can lower the acceptable quality limit of fresh fruit and vegetables, causing approximately one-third of these products to be thrown away [2]. FAO estimates that each year, approximately one-third of all food produced worldwide for human consumption is lost or wasted [3]. In Europe, for example, fruits and vegetables are the most wasted food products, representing 54% of total food loss and waste [4]. The economic impact of product losses is about 10% in Europe (6–7% in retailers) and 15% in the USA [5], while reaching 30% in developing countries (mostly due to a lack of temperature control). These product losses occur at all stages of the food supply chain. Temperature variations can occur during warehousing, handling and transportation [6]. Thus, the major challenge is to ensure a continuous cold chain from producer to consumer in order to guarantee the prime condition of goods [7].

It is not easy to maintain appropriate conditions throughout the entire chain, and negligence or mishandling in the logistics of perishable food products is very common and includes poor or excessive cooling of goods. Jedermann et al. reported many examples wherein inadequate temperature control management usually led to losses in the food chain (postharvest, distribution and home) [8].

Pelletier et al. carried out different temperature profiles and cooling procedures evaluating visual quality and weight loss as performance indicators for the different methods [9]. Apart from temperature, water loss is one of the main causes of deterioration that reduces the marketability of fresh fruits and vegetables. Free water, or condensation, is also a problem as it encourages microbial infection and growth and can also weaken packaging materials [10,11].

Some studies have demonstrated that there are significant RH variations within an individual cold room, which proves problematic for producing stability. It was demonstrated that the inside of a semi-trailer can have a broad dynamic RH range, from 55% (too dry) to 95% (too humid) [12]. However, it cannot be ignored that the ideal storage conditions for lettuce are far from ideal from those for tropical fruits, for instance, and would, in fact, cause chilling injuries to these products [13].

There are three types of heat transfer, according to Peck et al., that happen with passive thermal protection systems: conduction, convection and radiation. These explain how the heat is absorbed by the object (pallet covered or not). Conduction is the thermal energy transferred through matter; for example, the ground at high temperature transfers the heat to the pallet, and a protection that isolates the pallet from the ground would provide protection from temperature fluctuations. Convection is the thermal energy transferred through the motion of fluids; therefore, a pallet cover would protect the produce inside from the heat transferred by convection because it eliminates the air flow from the outside of the cover. Finally, radiation is the heat transfer by emission of electromagnetic waves; in this case, a cover that reflects part of that emitted energy would reduce the temperature increment [14].

Packaging effects in cold chain preservation have already been studied; Defraeye et al. presented a review about integrated performance for fresh produce packaging in the cold chain [15]. The study of the influence of pallet covers is not widely referenced in the literature, though Macnish et al. conducted some experiments studying the benefits of different cover systems over strawberry pallets. Presumably, the predominant heat transfer mechanisms that affect the loads are radiation and convection. The covers thus act as barriers to these two types of mechanisms and also as barriers to humidity transfer between the external environment and the air trapped between the covers and the product [16].

Different types of covers are evaluated in the present study and the different performance they offer regarding different products. In this case, an evaluation will be completed using thermal imaging since it has already been proven to be a powerful tool when working with real product scenarios, as some examples have demonstrated; Jha et al. discuss nondestructive quality evaluation methods for food and demonstrate how the cooked level inside a chicken can be assessed using thermal imaging and artificial neural networks (ANN) [17]. Chen et al. also demonstrated thermal imaging to be a technique for food safety, and Castro-Giraldez et al. implemented thermal imaging as a temperature control for the meat freezing process [18,19].

The main objectives of our study are to determine the suitability and compare the performance of the covers in real critical situations, such as potential exposure to temperatures outside of the acceptable range. It is expected that empirical data that demonstrate lower temperature loss compared with no coverage will be found.

## 2. Materials and Methods

### 2.1. Materials

An experimental refrigerated cold room, with programmable temperatures, was used to simulate a refrigerated condition during the produce cold chain. This has a polyurethane sandwich panel as isolation material, and the room measured 2 × 1.50 × 1.50 m. The refrigerated unit functions by internal evaporation with an external condensation unit of hermetic alternative compressor of 5 kW with R-134a as a refrigerant. Air speed is a constant, and it flows around 2.5 m/s. The room has its own temperature sensor to show the temperature in the control display. This sensor is placed on the wall of the door, 150 cm above the ground.

Data loggers were utilized to measure the ambient parameters. U12-013 HOBO measured temperature (range of −20° to 70 °C) and RH (5% to 95%). Each logger had two external ports where two temperature probes were connected, TMC6-HD and an air, water, or soil temperature sensor. The measurement range was −40° to 50 °C (−40° to 122 °F) in the water or soil and −40° to 100 °C (−40° to 212 °F) in the air.

The thermal camera utilized was Flir brand, E6 model. IR resolution was 160 × 120, with a thermal sensitivity of <0.006 °C, temperature range of –20° to 250 °C, and accuracy between ±2% or 2 °C.

**Figure 1:**
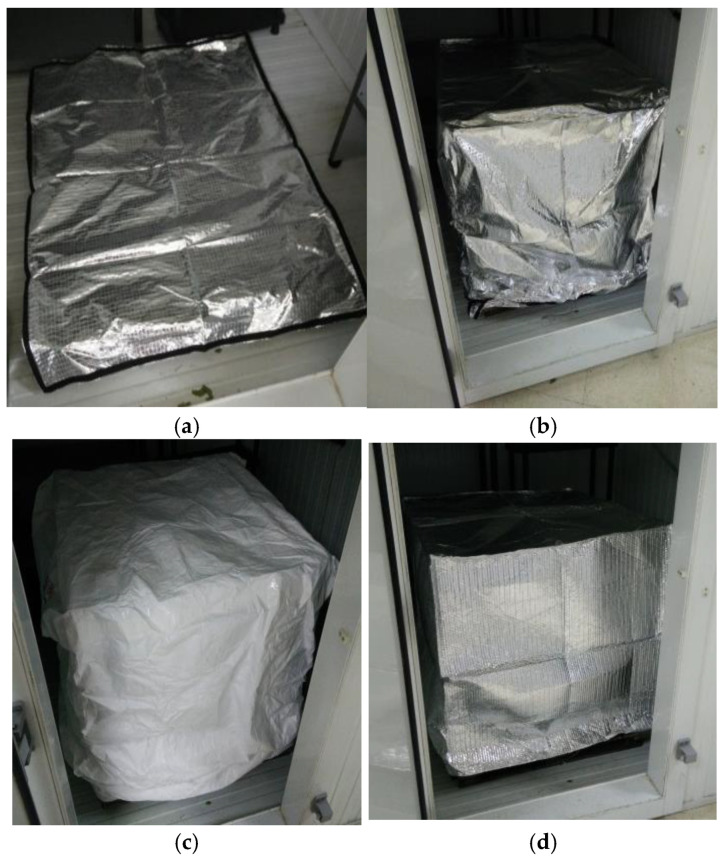
Covers placed on pallets: (**a**) Metalized PET base, (**b**) Metalized PET cover, (**c**) Tyvek^®^, (**d**) Metalized PET bubbles.

Three different types of pallet covers were used as coverage for the pallets (see Figure 1). The first was a DuPont™ Tyvek^®^ Air Cargo Cover, breathable, lightweight and made of Tyvek^®^, and 1200 × 1000 × 1700 mm in size. The second cover was made of Metalized PET and raffia and was not breathable; therefore, it accumulated condensation inside. The third was a cover composed of three layers, the inside and outside made of Metalized PET with the middle layer made of plastic bubbles. The size of the last two covers was 1200 × 1000 × 1700 mm. The pallet used was 1200 × 1000 mm in size, and the plastic grid boxes were 500 × 380 × 280 mm.

The products used were fresh vegetables, and in all cases, the experiment did not start more than 24 h after harvest. Products included 400 kg of chard for the first experiment, 400 kg of cucumbers for the second, and 500 kg of carrots for the last experiment.

The optimum storage conditions for these products are presented in Table 1. As can be seen, carrots can be stored for a longer period of time than chard or cucumbers due to their lower water content.

### 2.2. Methods

IR camera cannot give a surface temperature with precision, and thus calibration is necessary. Prior to the experiment, calibration of the thermal camera was carried out due to the emissivity of the materials in the covers tested. That calibration was made by preparing a flat surface of the covers, attaching a TMC6-HD probe to it and measuring the temperature. Then, a thermal picture was taken in the probe area, the temperature from the probe and the IR image were compared, and the emissivity was adjusted in the camera settings.

The following method descriptions are separated according to the three different products used. The three products combined with the different covers have different experimentation times but share the same procedure.

Each product was distributed into 18 boxes—about 15 kilos per box for chard and 20 kilos for cucumbers and carrots.

**Figure 2:**
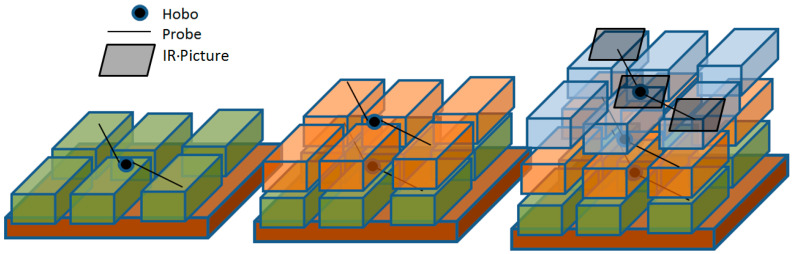
Sensor distribution in the pallet.

The pallet was built in three layers, with a data logger installed in the center of each layer of the boxes and two probes also installed inside the boxes at the end of a cable in opposite boxes diagonally, as shown in Figure 2. In total, there were 3 humidity sensors in the middle of the three layers and 9 temperature sensors, conforming to a diagonal mesh of sensors in the pallet. The sensors were set to record temperature and humidity every 5 min. The position of the sensors covers the three highs of the pallet and one diagonal, which represents the maximum, minimum and average temperatures among the pallet, as observed in previous studies [21,22,23].

In the following step, three pictures from the surface on top of the pallet were taken using the thermal camera, one picture from the top-right corner, one in the middle and one in the bottom-left corner, as indicated in Figure 2. On top of the pallet representation, the three pictures taken are represented—one in the upper-left part of the image, the other in the middle and the other in the bottom-right. These pictures are intended to be used as a complement to the study; the pictures provide the produce surface temperature, which is the temperature over the entire pallet since the product was not exposed to any heat/cold source. The pallet was then covered with one of the types of covers.

**Figure 3:**
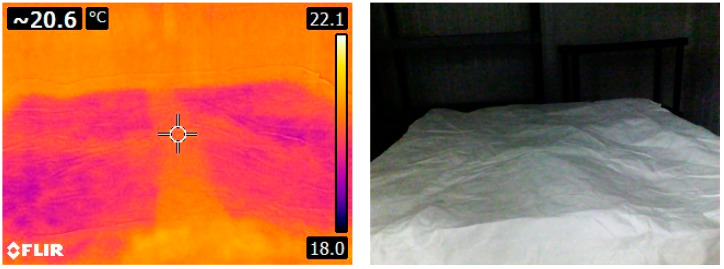
IR image from the pallet surface covered with Tyvek^®^ cover.

At this moment, the product was at room temperature and the cold room was set to cool down to a temperature of 4 °C; five pictures were taken with the IR camera, specifically the four top corners and the middle of the pallet’s upper face center. After 24 h, the refrigerating unit was switched off to simulate a failure in the cooling system. The temperature increased until it reached the ambient temperature (23 °C), and the surface of the pallet was recorded with pictures from the thermal camera and the corresponding temperature data that was registered in the image; those pictures were taken at the same points as in the beginning, the four top corners and the pallet’s top face center. The aim of taking those pictures was to have a record of the critical moments, such as the extreme temperatures at the beginning and at the end of the dynamic temperature curves. The temperature from the IR image was extracted and used in the results (Figure 3).

The temperature ramp from 4 °C to 10 °C represents an important gap that could affect the quality of the vegetables. This leap is common when there is a break in the cold chain, such as refrigeration failure or when the freight is exposed to ambient temperatures; the product temperature might not reach the ambient temperature, but it increases. In addition, the authors wanted to have identical temperature ranges in all the scenarios, which makes the experiments replicable.

Thus, the temperature ramp was replicated twelve times, with three different real fresh vegetables—it is important to remark that real produce is a difficult-to-find good in research experiments due to their perishable characteristics—and with each vegetable type, it was repeated using three different covers and no cover.

When the process was finished, the data were analyzed with ad hoc software developed in MATLAB 2022a. The correlation between the thermal images and sensor temperatures was studied by conducting a statistical analysis. RMSE (Root Mean Squared Error) was used to obtain an error with the same unit as the outcome variable for easier interpretation purposes. The closer the point is to the regression, the lower the metric value and the higher the accuracy of the regression model. When a model is 100% perfect, this metric value will be equal to zero. The RMSE formula is

(1) $$RMSE=\sqrt{\frac{1}{N}\times \sum_{i=1}^{N}{\left(y_{i}-\hat{y_{i}}\right)}^{2}}$$

where

N is the number of samples;

Y_i_ are the observed values;

ŷ_i_ are the estimated values.

## 3. Results

The presented results are separated by product. Independent figures and tables are shown for each kind of product.

### 3.1. Chard

**Figure 4:**
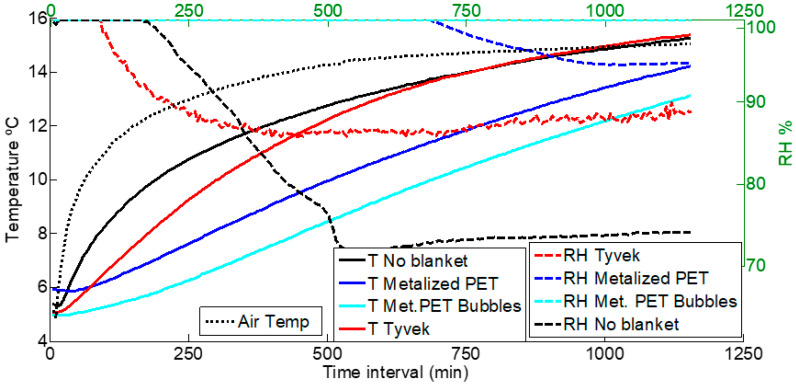
Comparison of different cover temperatures and RH for chard.

Figure 4 shows the temperature lines following a rising trend for a pallet of chard; the dotted line represents the temperature changes in the air of the cold room, and the solid lines represent an average (from the nine temperature sensors) of the air temperatures inside the pallet. 

The Metalized PET bubble cover provided the slowest temperature increase, followed by the Metalized PET cover; the two are closer than they appear in the graph because the start point of the Metalized PET cover was approximately one degree Celsius above the Metalized PET bubble cover.

On the other hand, the Tyvek^®^ cover presents a more inclined line than the other two covers. In the beginning, this line stays below the no-cover temperature, but after 750 min (12.5 h), the two lines merge until the end of the graph. Both lines merge with the air temperature after 1000 min.

The dashed lines in Figure 4 represent the relative humidity measurements during the chard experiment; these are plotted versus the temperature lines in order to compare the influence of both parameters simultaneously. In the case of no cover, the fall of RH is almost instant. It is important to note that because the water content in chard leaves is very high, the humidity loss is high as well; therefore, the sensors encapsulated in the plastic covers were easily saturated with water, and the fall of the RH lines may possibly present some delay with regard to the actual humidity. With the Tyvek^®^ cover, the RH fell after 200 min and remained stable at around 90% for the rest of the time. For the no-cover scenario, the RH line presents a downward trend from the time interval from 200 to 500 min, but in this case, the RH reached 75%. The Metalized PET bubble cover did not present any drop throughout the duration of the experiment, and for the Metalized PET cover, RH only fell after 700 min, and then it never fell below 95%.

Among all the times measured by the sensors installed in the pallet, the maximums, minimums and means are represented in Table 2. The time for the temperature to increase from 4 to 10 °C presents an upward trend starting with no cover, then Tyvek^®^, Metalized PET and, finally, Metalized PET bubbles in Table 2 for the maximum. The Metalized PET and Metalized PET bubbles switch positions for the average and minimum times for the temperature increase.

The overall maximum is found with the Metalized PET cover and the minimum during the no-cover experiment. The average times are very close in the Tyvek^®^ and no-cover scenarios, which present concordance with Figure 4, where it can be observed that the two curves come closer together at the end of the curve.

**Figure 5:**
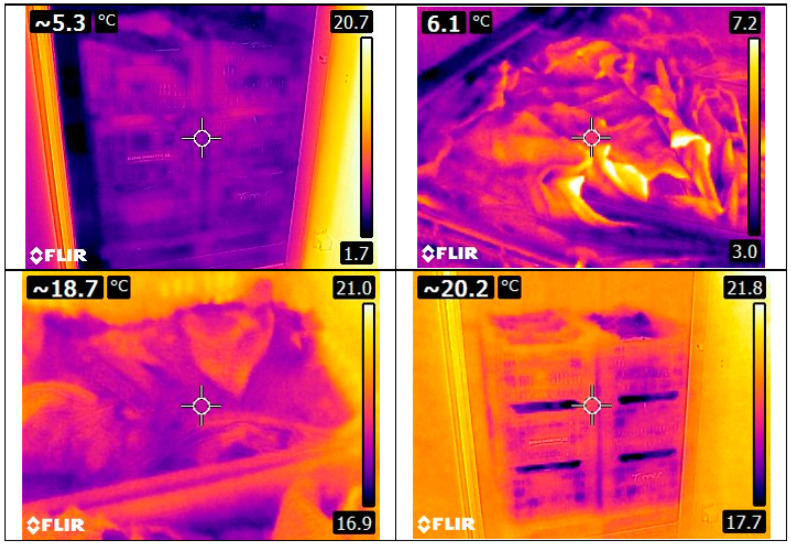
Some thermal images of the pallet with chard and without cover.

As explained in the Methods Section, thermal images (see Figure 5) were taken at specific times, and the temperatures were compared with all the temperature points, resulting in the Root Mean Square Error (RMSE) between the sensor temperature and the thermal image temperature (see Table 3). The maximum error corresponds to the Metalized PET bubble cover, but on the other hand, it presents the minimum error as well. 

### 3.2. Cucumbers

In the case of the cucumbers, the temperature increase shortened over time, reaching 350 min while the chard and carrots surpassed 1000 min.

**Figure 6:**
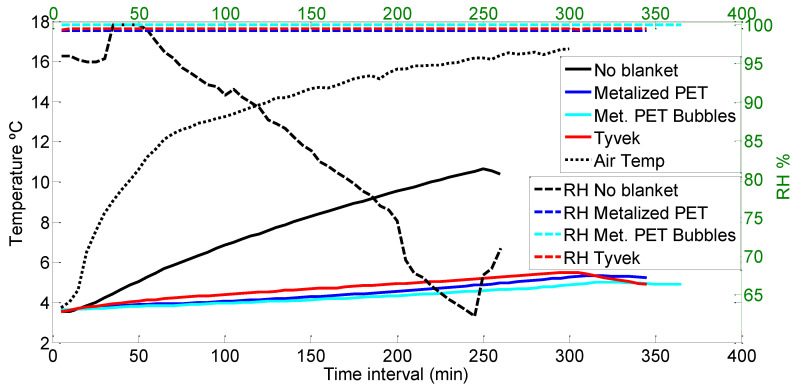
Comparison of different cover temperatures and RH for cucumbers.

Figure 6 shows how the air temperature increased faster than the other temperatures and reached a certain stabilization point around 75 min. The following temperature upon the increase is obtained during the no-cover experiment. All the covers presented a very small increase during 350 min, barely reaching 5 °C, with the Tyvek^®^ being the fastest to increase, followed by the Metalized PET and, finally, the Metalized PET bubble cover.

The relative humidity, in this case, did not fall at any moment and remained at 100% for the three different covers, with the only exception in the graph being the no-cover RH line, which decreases rapidly when the temperature rises. This happened drastically 75 min after the temperature increase began (see Figure 6).

It was not possible to achieve a temperature increase from 4 °C to 10 °C for all of the covers due to some technical problems, and therefore, some spaces remain blank in Table 4. Between the Tyvek^®^ and the no-cover situations, Tyvek^®^ gives longer times in the means and in the maximum, but for the minimum, the situation is reversed, and no cover was 15 min slower than the Tyvek^®^ cover.

**Figure 7:**
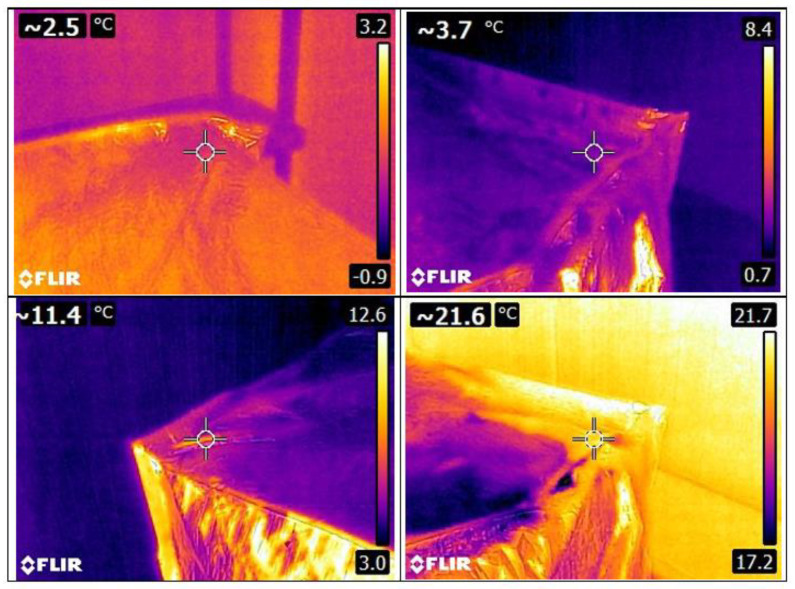
Some thermal images of the pallet with cucumber and Metalized PET cover.

Finally, in the cucumber section, the differences between the temperature given by the camera (see Figure 7) and the pallet are shown in Table 5. The maximum difference occurred when using the Metalized PET bubble cover with an RMSE of 21.8 °C; this cover also had the second lowest error, 0.3 °C, with the lowest error being the Metalized PET cover (0.1 °C). The biggest difference between errors is in the maximum line, or the 4.3 °C of difference, when comparing the Metalized PET maximum and the Metalized PET bubbles maximum.

### 3.3. Carrots

**Figure 8:**
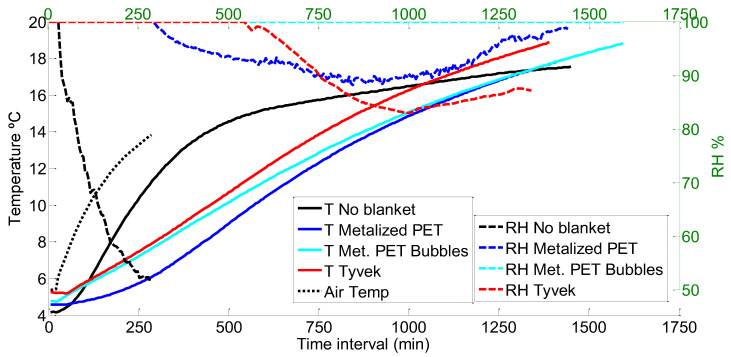
Comparison of different cover temperatures and RH for carrots.

Figure 8 shows how the temperature increased in the carrot pallet with the different covers. All the measurements start from around 4 and 5 °C and present an upward trend according to the temperature increase in the room. The air temperature, as expected, was the fastest temperature to rise, and the no-cover temperature was the second fastest. In this case, the three covers presented more similar results than for the chard pallet, which will be reflected in Table 6, as well. The fastest line to rise from the covers is, as in the other two examples, the Tyvek^®^, followed by the Metalized PET bubbles and the Metalized PET coming in last.

The temperature increase produced an RH decrease (see Figure 8); the no-cover scenario was the fastest scenario to lose humidity, reaching almost 50% RH in barely 250 min. Then, the Metalized PET cover presented a decrease in humidity to 90%, but only after more than 875 min, very similar to the Tyvek^®^ cover, yet the RH remained at 100% for a longer time and descended to 85% at the end of the curve. The Metalized PET bubble cover, on the other hand, did not decrease the humidity at any moment, creating condensation inside the pallet.

In this case, the time to reach 10 °C from 4 °C for all the covers was very similar between the three (see Table 6); the mean time rose from 408 to 450 min, while the time to reach 10 °C with no cover was shorter (162 min). In the maximum, the biggest difference is found between the Metalized PET bubbles and the Metalized PET (105 min). Finally, the minimum times were again more homogeneous, with the Tyvek^®^ and the Metalized PET bubble covers being closer than the Metalized PET.

**Figure 9:**
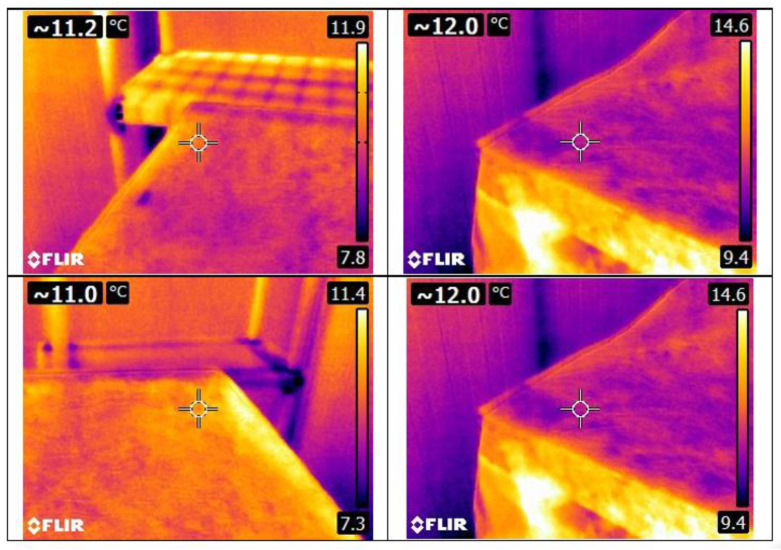
Thermal images of the pallet with carrots and Metalized Tyvek^®^ cover.

Table 7 is the comparison of carrots of the thermal camera (see Figure 9) versus the pallet temperature. The biggest RMSE is given with the Metalized PET bubble cover reaching 15.6 °C, and similar to the chard experiment, the smallest RMSE also occurred with the bubble cover. The Tyvek^®^ cover presented the lowest mean and maximum errors, and the Metalized PET cover had the highest error from the minimum error line.

**Figure 10:**
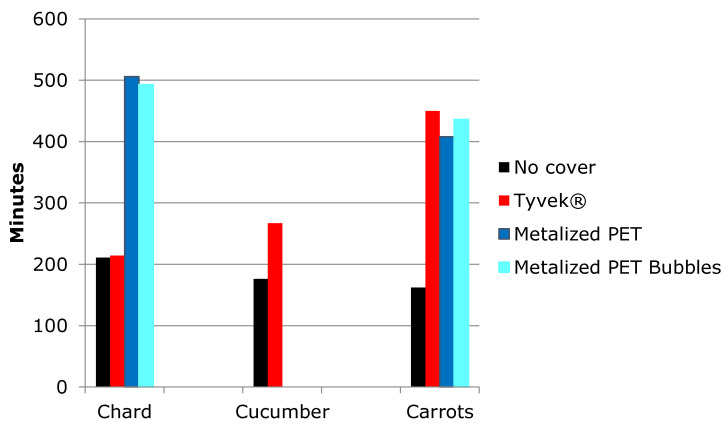
Time to rise from 4 °C to 10 °C (average).

Finally, Table 8 summarizes the results of all the replications, including the standard deviation of mean, maximum and minimum temperature. There was more dispersion in the results with Tyvek cover and less with no cover. Metalized PET with bubbles produced the best results. And in Figure 10, the times to rise from 4 to 10 °C are displayed in a columns figure, which gathers the average times from Table 2, Table 4 and Table 6. The times for no cover in the three products are similar, from 211 (chard) to 162 (carrots) minutes; the times for Tyvek^®^ present the greatest changes among different products, from 214 (chard) to 450 (carrots). And the two Metalized PET covers do not present a variability as big as Tyvek^®^ between chard and carrots. As stated before, it was not possible to achieve the temperature rising times from Metalized PET covers due to technical problems.

## 4. Discussion

The minimum increase times were the most important since this was when some parts of the pallet started to increase in temperature to critical points. All the cases had a better performance or slower temperature rising times in the transition from 4 to 10 °C when comparing cover versus no cover, with the only exception being the cucumbers, for which the Tyvek^®^ and no-cover values were very close (30 and 45 min, respectively). These minimum times always occur in the top layers of the pallet, a fact that can indicate the covers could be enhanced in the top part, for example, by adding an extra layer of material just at the top.

The Tyvek^®^ cover was demonstrated to attenuate the effect in the thermal increment and presents lower condensation rates than the other two covers. Peck et al. showed that single-skin fabric covers could provide resistance to certain temperature changes when a hot or cold air stream happens; if these fabric covers are permeable, they provide breathability and, therefore, can avoid or lower condensation. Also, the studied cover made of Metalized PET presents an acceptable attenuation for thermal increment, but the breathability is lower than in the case of Tyvek^®^. However, as explained by Peck et al. (2015), the reflective material of this cover is expected to be effective against radiation, especially direct sunlight. With the Metalized PET bubble cover, it is expected to have a slower rate of thermal change due to the thicker insulating character of the material, and as shown in the results, slower temperature changes occur using the Metalized PET bubble cover [14].

In terms of humidity, in the case of chard, the humidity lines fell faster in the beginning for Tyvek^®^, but at the end, RH was the lowest with no cover, reaching 75%, while Tyvek^®^ never fell below 90%. In the other two products, no-cover RH always fell faster, though in reality, for the cucumbers, none of the lines for the covers fell at all; however, this is attributed to the short experiment time. According to Table 1, the most suitable cover for all the products (with regard to RH) would be the Metalized PET cover because, despite the Metalized PET bubble cover never presenting any fall in RH, it caused significant condensation inside the pallet. This condensation could ultimately cause mold or fungus problems.

Differences between products with the same covers in terms of temperature and RH were also found. With regard to temperature increment times, a wide range between chard and carrots in Tyvek^®^ was found. And regarding RH, a completely different behavior was found for the Metalized PET cover among the three scenarios (chard, cucumber and carrots). It could be caused by their different physiological characteristics, which should be studied in future research.

As pointed out in the literature [24], the emissivity of the surfaces may cause difficulties during the interpretation of the thermograms; a reflected image appears when there is a radiation source that directly impacts the surface of the covered pallet and can create a “false positive”. Despite this, the IR results are consistent with those obtained with the sensors, and the best results were obtained with the lights off inside the cold room.

## 5. Conclusions

In light of the results, it is clear that pallet covers are very useful in case of a cold chain break or malfunction. Pallet covers are able to prevent conduction, convection heat transfer and provide insulation for the perishable food products studied. In case of refrigeration failure during the cold chain, the protective barrier keeps products within an acceptable temperature range for hours. The different types of covers evaluated in the present study show temperature preservation with regard to no cover. The temperature changes occur slower than with no cover. The empirical data demonstrate lower temperature loss compared with no coverage.

When comparing covers, the Metalized PET bubble cover shows better protection than the other two covers. The Tyvek^®^ cover shows the lowest isolation rates. The temperature increments follow an expected order, especially in cucumbers and carrots, where the pallets covered show a temperature rise clearly different (slower) than no cover.

On the other hand, with no cover, the RH always falls below any cover scenario, depending on the product, and also, there are differences observed between different products for the covers. With Tyvek^®^, the RH falls in chard and carrots (Metalized PET also falls with both products), but in carrots, it falls faster than in chard and, towards the end, presents a rising trend. The influence of the different products in the different blankets is a fact that was revealed in the results of this work, but it is not the subject of study in this document; though it should be studied in depth in future experiments, and the reason why they cause different RH and temperature trends should be explained.

The evaluation with the thermal camera was a difficult task to carry out since the average errors ranged from 4.1 to 8.8 °C. These are not good indicators of what is happening below the cover and even less so in the middle of the pallet. However, the minimum errors ranged between 0.05 and 1.6 °C, which could be promising data for further studies. 

## Figures and Tables

**Table 1:** Storage conditions for vegetables [20].

Storage Conditions			
Vegetable	Temperature (°C)	RH (%)	Storage Life
Chard	0	95–100	10–14 days
Cucumber	10–12.78	95	10–14 days
Mature Carrot	0	98–100	7–9 months

**Table 2:** Time to increase from 4 °C to 10 °C (min) (chard).

Chard Initial Point 4 °C	No Cover	Tyvek^®^	Metalized PET	Metalized PET Bubbles
Mean time (min)	211	214	506	493
Max. time (min)	355	560	745	675
Min. time (min)	30	40	75	70

**Table 3:** RMSE between the thermal image and sensor temperature (Chard).

Chard	Tyvek^®^	Metalized PET	Metalized PET Bubbles
Mean temperature (°C)	7.2	4.2	5.5
Max. temperature (°C)	14.1	12.2	16
Min. temperature (°C)	1.6	0.2	0.1

**Table 4:** Time to increase from 4 °C to 10 °C (min) (Cucumber).

Cucumber Initial Point 4 °C	No Cover	Tyvek^®^	Metalized PET	Metalized PET Bubbles
Mean time (min)	176	267	--	--
Max. time (min)	290	480	--	--
Min. time (min)	45	30	--	--

**Table 5:** RMSE between the thermal image and sensor Temperature (Cucumber).

Cucumber	Tyvek^®^	Metalized PET	Metalized PET Bubbles
Mean temperature (°C)	5.5	4.1	5.8
Max. temperature (°C)	18.5	17.5	21.8
Min. temperature (°C)	1.23	0.1	0.3

**Table 6:** Time to rise from 4 °C to 10 °C (min) (Carrots).

Carrot Initial Point 4 °C	No Cover	Tyvek^®^	Metalized PET	Metalized PET Bubbles
Mean time (min)	162	450	408	436
Max. time (min)	250	620	670	565
Min. time (min)	30	150	80	135

**Table 7:** RMSE between the thermal image and sensor temperature (Carrots).

Carrots	Tyvek^®^	Metalized PET	Metalized PET Bubbles
Mean temperature (°C)	6.2	6.9	8.8
Max. temperature (°C)	10.7	12.1	15.6
Min. temperature (°C)	0.1	1.6	0.05

**Table 8:** Summary of results. Time to increase from 4 °C to 10 °C, including standard deviation.

Initial Point 4 °C	No Cover	Tyvek^®^	Metalized PET	Metalized PET Bubbles
Mean time and standard deviation (min)	183 ± 25	310 ± 124	457 ± 69	465 ± 40
Max. time and standard deviation (min)	298 ± 53	553 ± 70	708 ± 53	620 ± 78
Min. time and standard deviation (min)	35 ± 9	73 ± 67	78 ± 4	103 ± 46

## Data Availability

The data used to support the findings of this study can be made available by the corresponding author upon request.
